# Rural and Appalachian cancer survivors’ responses to tobacco use screening and tobacco treatment offer

**DOI:** 10.18332/tid/207082

**Published:** 2025-09-04

**Authors:** Abigayle R. Feather, Brent J. Shelton, Courtney Blair, D. Bront Davis, Joan Scales, Audrey Darville, Joseph Valentino, Laurie E. McLouth, Jessica L. Burris

**Affiliations:** 1 Department of Psychology, College of Arts and Sciences, University of Kentucky, Lexington, United States; 2 Markey Cancer Center, University of Kentucky, Lexington, United States; 3 Department of Internal Medicine, College of Medicine, University of Kentucky, Lexington, United States; 4 College of Nursing, University of Kentucky, Lexington, United States; 5 Department of Otolaryngology-Head and Neck Surgery, College of Medicine, University of Kentucky, Lexington, United States; 6 Department of Behavioral Science, College of Medicine and Center for Health, Engagement, and Transformation, University of Kentucky, Lexington, United States

**Keywords:** tobacco use cessation, cancer survivors, rural population, patient acceptance of healthcare

## Abstract

**INTRODUCTION:**

Smoking after a cancer diagnosis is associated with poor outcomes whereas smoking cessation improves survival and other outcomes. Although professional societies and practice guidelines call for equitable tobacco treatment delivery in healthcare, disparities in tobacco-related disease burden persist.

**METHODS:**

In the context of an outpatient US cancer center’s population-based tobacco treatment program, this study examines associations between cancer survivors’ rural and Appalachian residence status and: 1) current tobacco use status, 2) decision to decline tobacco treatment, and 3) reason for declining assistance. A cross-sectional, retrospective analysis was conducted using electronic health record data from 16839 adults: 64.04% female, 88.49% non-Hispanic White, mean age 59.19 ± 14.52 years, 35.97% rural residence, 53.14% Appalachian residence, who sought cancer care in 2019. Descriptive statistics and logistic regression models were applied.

**RESULTS:**

The portion of patients that reported current tobacco use was 21.75%. Rural patients had higher odds of tobacco use than urban (OR=1.22; 95% CI: 1.12– 1.34), as did Appalachian patients compared to non-Appalachian (OR=1.41; 95% CI: 1.28–1.54). Neither rural nor Appalachian residence status was significantly associated with responses to tobacco treatment offers (76.65% declined the offer) or reason for declining (65.19% declined because they were ‘not ready to quit’).

**CONCLUSIONS:**

Findings highlight continued need for population-level tobacco use screening and proactive tobacco treatment offers, given elevated tobacco use in some minority groups and overall low rates of tobacco treatment acceptance. This large study helps shed light on the association between geographical residence and tobacco-related outcomes among patients with cancer, and underscores room for improvement in tobacco treatment uptake in cancer care.

## INTRODUCTION

Cancer is a major public health concern, with over two million new cases in the United States (US) projected for 2024^1^. Among the general US population, the lifetime risk of developing cancer is currently one in three^2^, with the most common disease sites being breast, colorectal, lung, and prostate. Advances in early detection and treatment have led to significant reductions in cancer mortality, with a 33% overall reduction since 1991. Five-year survival rates have increased from 49% in 1975 to 68% in 2018, leading to more and more people living well beyond their cancer diagnosis^2^. Indeed, there are more than 18 million US cancer survivors today, a number that could reach 26 million by 2029^2^.

As cancer treatments improve and survival rates rise, secondary disease prevention and health promotion among cancer survivors are paramount. Individuals with a cancer history are at high risk of developing other chronic conditions such as hypertension, pulmonary diseases, and diabetes^3^. They also tend to experience high rates of chronic or recurring sleep problems, fatigue, distress, and pain^4^. Poor overall health among cancer survivors is influenced by factors such as healthcare access and quality, as well as the side- and late-effects of cancer treatments^5^. Another contributing factor is the increased prevalence of unhealthy behaviors (e.g. heavy alcohol use, cigarette smoking, sedentary behavior) among cancer survivors compared to those without a cancer history^6^. To facilitate disease prevention and health promotion, professional organizations have established and disseminated national guidelines on health behaviors to cancer survivors and their cancer care teams^7^, with one common recommendation being abstinence from tobacco use.

Tobacco use abstinence is critically important for cancer survivors, as it can improve and extend their lives. The 2014 US Surgeon General’s Report concluded a causal relationship between cancer survivors’ smoking and all-cause mortality, cancerspecific mortality, and second primary cancer^8^. Smoking is also associated with an increased risk of cancer recurrence, poorer response to cancer treatment, and higher cost of cancer care^8,9^. Additionally, cancer survivors who smoke are more likely to report greater symptom burden during routine care, experience surgical site infection and other complications, and return to the operating room more than their non-smoking counterparts^10^. Importantly, there is ample empirical evidence that smoking cessation after cancer diagnosis is associated with reduced overall and cancer-specific mortality^8,11^ plus other health benefits^12^. Despite all this, 12–33% of cancer survivors report current cigarette smoking or other tobacco use^13^.

While quality cancer care should be accessible to, affordable for, and provided to everyone, there are well known inequities in cancer care delivery such that historically marginalized groups often bear the brunt of adverse outcomes. These iniquities are evident across various factors, including race, ethnicity, English proficiency, insurance coverage, sexual and gender identity, and geographical residence^14^. Not discounting the multitude of other sociodemographic factors linked to cancer care inequities, the focus of the current study is geographical residence. National surveys indicate that 21% of cancer survivors live in rural areas, amounting to nearly 3 million individuals, a considerable segment of the US population^15^. Critically, rural areas have seen slower reductions in cancer incidence and mortality^16^. Additionally, significant differences in tobacco use and cessation rates exist between the US rural and urban population, with 26% of rural residents reporting current tobacco use compared to 18% of urban residents^17^. Appalachia – a geographically distinct and socioeconomically diverse region that spans parts of 13 US states – faces persistent health disparities and markedly higher rates of smoking than the national average (33%), challenges that extend beyond those seen in other rural areas^18^. Given the heightened cancer burden in rural and Appalachian populations and the significance of tobacco use after cancer diagnosis, it is important to determine rural-urban and Appalachian–non Appalachian differences in cancer survivors’ responses to proactive, populationbased offers of tobacco treatment. This study aims to investigate how rural and Appalachian residence relate to tobacco use and treatment engagement among cancer survivors within a population-level tobacco treatment program.

### Current study

Within the context of a newly implemented tobacco treatment program at an outpatient cancer care facility, the current study examined whether geographical residence was independently associated with current tobacco use, declining an offer of tobacco treatment, and reasons for declining tobacco treatment. Based on prior literature we hypothesized that rural and Appalachian residence status would have a significant, negative effect across all outcomes, such that cancer survivors from rural and Appalachian areas (examined separately) would be more likely to report current tobacco use, decline tobacco treatment, and decline treatment specifically because they are not ready to quit. The rationale for studying Appalachian residence in addition to rural residence is that while the Appalachian region faces similar challenges to rural areas, it is a unique geographical area of historical independence and resilience, with health disparities in Appalachia especially pronounced in part due to its well-documented social disadvantages, history of economic exploitation, and increased environmental exposure^19^.

## METHODS

### Study design, setting, and sample

This cross-sectional, retrospective study was conducted using electronic health record data from a National Cancer Institute (NCI)-designated comprehensive cancer center located centrally within a southeastern US state. The cancer center is in an urban county, but its catchment area is the entire state, with many patients traveling from rural and Appalachian areas to seek care. The study population includes all adults (aged ≥18 years) who presented for outpatient care between 1 January 2019, and 31 December 2019. The final analytic sample (n=16839) was restricted to individuals with complete data on residential status (loss of 40 cases) and tobacco use status (loss of 10 cases).

### Procedures

As part of standard care, the cancer center’s rooming procedures involve clinical service technicians asking standardized tobacco use screening questions and recording patients’ responses to these questions in the electronic health record. This study is a retrospective review of patients’ responses plus selected demographic and clinical variables extracted from the electronic health record. All data were de-identified and aggregated for the purpose of analysis. Research procedures were ruled exempt by the Institutional Review Board of the University of Kentucky (Protocol 52059).

### Measures

*Background characteristics*

Patients’ demographic information pertinent to age, sex, race, ethnicity, marital status, and insurance type were recorded using a fixed set of response options. The clinic where patients were seen is a proxy for disease site, with breast, gynecology, and hematology as disease-specific clinics and the multidisciplinary clinic being the location where patients with other cancers (e.g. larynx, colorectal, bronchus) were seen. Self-reported level of distress in the past week was reported on a scale from 0=no distress to 10=extreme distress^20^, and is considered a clinical variable.

*Rural and Appalachian residence*

Patients’ county of residence was used to measure, as separate variables, rural and Appalachian residence status. For rurality, the latest US Rural-Urban Continuum Codes (RUCC) were used, codes which classify counties by population size and proximity to metropolitan areas. RUCC range from 1=counties in metropolitan areas of 1 million or more residents to 9=completely rural or less than 2500 urban population, not adjacent to a metropolitan area^21^. The continuum was dichotomized such that codes 1–6 represent urban areas and codes 7–9 indicate rural residence, in order to focus the analysis on those who are most likely face geographically driven disparities in health outcomes^22^. County of residence was also used to create a dichotomy of Appalachian or non-Appalachian residence status according to the US Appalachian Regional Commission^23^.

*Tobacco use and related outcomes*

Patients were asked if they have ever used tobacco (yes/no), and those who reported lifetime tobacco use were asked about the types of tobacco products used (e.g. cigarettes, cigars/pipes), and the last time they used tobacco (e.g. today, more than one year ago), both of which have fixed response options. Standard clinical protocol dictates that all patients who report current tobacco use (i.e. past 30 days) are then asked if they would like help quitting (yes/no) consistent with a proactive offer of tobacco treatment. Patients who decline the offer of tobacco treatment (which would have begun with an individual meeting with a certified tobacco treatment specialist to develop a personalized, evidence-based treatment plan) were asked the reason for their referral decline, again with fixed response options (specifically, ‘already in treatment’, ‘want to quit on my own’, or ‘not ready to quit’). The three outcome variables are: 1) current tobacco use status, 2) tobacco treatment engagement response, and 3) reasons for declining treatment.

### Data analysis

Descriptive statistics (e.g. percentages, frequencies) were used to describe the sample’s background characteristics, residential status, and tobacco use and related outcomes. Binomial (current tobacco use and tobacco treatment response) and ordinal logistic regression (reasons for declining) were conducted to assess the independent influence of residential status across all outcomes, with rural and Appalachian residence tested in separate models. A prior, similar study found multiple background demographic and clinical variables were associated with one or more of the outcomes of interest^13^, so sex, age, race and ethnicity, marital status, insurance status, clinic and distress level were all treated as covariates in the models tested here. Statistical significance was set at p<0.05 in all analyses and the assumptions for all models, including proportional odds, were checked. Adjusted odds ratios (AORs) and corresponding 95% confidence intervals (CIs) were calculated for all model estimates. Model goodness of fit for binary logistic regressions was assessed using the Hosmer-Lemeshow (H-L) test. All hypothesis tests were two-tailed, and all statistical analyses were conducted in SAS version 15.2 (SAS Institute, Cary, NC) and SPSS version 28.0 (SPSS Inc., Chicago, IL).

## RESULTS

### Sample characteristics

Tables 1 and 2 provide for full details on the sample’s background. Patients had a mean age of 59.19 ± 14.52 years. There were more female (64.04%) than male patients and most patients were White, non-Hispanic (88.49%). Most patients were either currently married (55.87%) or widowed, separated or divorced (23.26%). Nearly all patients had insurance coverage, with most having Medicare (44.27%) or Medicaid (18.05%). Patients were seen in these outpatient clinics: multidisciplinary (48.04%), hematology (17.99%), breast (17.03%), and gynecological (16.94%). Patients reported mild distress levels (range: 0–10), on average (3.27 ± 3.12). Regarding geographical area of residence, 35.97% of patients were categorized as having rural residence, and just over half lived in Appalachia (53.14%).

**Table 1:** Demographic and clinical characteristics of US cancer survivors seeking care at a cancer center, by residence status, a cross-sectional analysis, 2019 (N=16839)

Characteristics	Total (N=16839) % (n)	Rural ^a^ (N=5905) % (n)	Urban (N=10934) % (n)	p
**Sex**				0.387
Female	64.04 (10783)	64.47 (3807)	63.80 (6976)	
Male	35.96 (6056)	35.53 (2098)	36.20 (3958)	
**Race and ethnicity**				<0.001
Racial and/or ethnic minority	8.20 (1381)	1.78 (105)	11.67 (1276)	
White, non-Hispanic	88.49 (14901)	94.65 (5589)	85.17 (9312)	
Missing	3.31 (557)	3.57 (211)	3.16 (346)	
**Marital status**				<0.001
Married	55.87 (9408)	57.14 (3374)	55.19 (6034)	
Single/never married	17.77 (2992)	14.77 (872)	19.39 (2120)	
Widowed/separated/divorced	23.26 (3916)	24.52 (1448)	22.57 (2468)	
Missing	3.11 (523)	3.57 (211)	2.85 (312)	
**Insurance status**				<0.001
Managed care organizations	34.95 (5885)	28.02 (1708)	38.20 (4177)	
Medicaid	18.05 (3040)	21.68 (1280)	16.10 (1760)	
Medicare	44.27 (7454)	47.52 (2806)	42.51 (4648)	
Self-pay or other	2.73 (460)	1.88 (111)	3.19 (349)	
**Age** (years), mean ± SD	59.19 ± 14.52	59.35 ± 13.95	59.10 ± 14.82	0.029
**Clinic**				<0.001
Breast	17.03 (2868)	14.77 (872)	18.25 (1996)	
Gynecology	16.94 (2853)	21.12 (1247)	14.69 (1606)	
Hematology	17.99 (3029)	13.84 (817)	20.23 (2212)	
Multidisciplinary	48.04 (8089)	50.28 (2969)	46.83 (5120)	
**Distress** (0–10), mean ± SD	3.27 ± 3.12	3.45 ± 3.16	3.17 ± 3.09	<0.001^b^

a Rural refers to a definition of ‘rural’ as inclusive of RUCC 7-9.

b Mann-Whitney test used due to non-normal distribution of variable.

**Table 2:** Demographic and clinical characteristics of US cancer survivors seeking care at a cancer center, by Appalachian residence status, a cross-sectional analysis, 2019 (N=16839)

Characteristics	Total (N=16839) % (n)	Appalachian (N=8948) % (n)	Non-Appalachian (N=7891) % (n)	p
**Sex**				0.605
Female	64.04 (10783)	64.22 (5746)	63.83 (5037)	
Male	35.96 (6056)	35.78 (3202)	36.17 (2854)	
**Race and ethnicity**				<0.001
Racial and/or ethnic minority	8.20 (1381)	1.94 (174)	15.30 (1207)	
White, non-Hispanic	88.49 (14901)	94.60 (8465)	81.56 (6436)	
Missing	3.31 (557)	3.45 (309)	3.14 (248)	
**Marital status**				<0.001
Married	55.87 (9408)	57.66 (5159)	53.85 (4249)	
Single/never married	17.77 (2992)	14.54 (1301)	21.43 (1691)	
Widowed/separated/divorced	23.26 (3916)	24.42 (2185)	21.94 (1731)	
Missing	3.11 (523)	3.39 (303)	2.79 (220)	
**Insurance status**				<0.001
Managed care organizations	34.95 (5885)	30.17 (2700)	40.36 (3185)	
Medicaid	18.05 (3040)	20.37 (1823)	15.42 (1217)	
Medicare	44.27 (7454)	47.38 (4240)	40.73 (3214)	
Self-pay or other	2.73 (460)	2.07 (185)	3.48 (275)	
**Age** (years), mean ± SD	59.19 ± 14.52	59.51 ± 14.05	58.83 ± 15.03	
**Clinic**				<0.001
Breast	17.03 (2868)	14.87 (1331)	19.48 (1537)	
Gynecology	16.94 (2853)	19.90 (1781)	13.59 (1072)	
Hematology	17.99 (3029)	13.92 (1246)	22.60 (1783)	
Multidisciplinary	48.04 (8089)	51.30 (4590)	44.34 (3499)	
**Distress** (0–10), mean ± SD	3.27 ± 3.12	3.38 ± 3.15	3.15 ± 3.01	<0.001^a^

a Mann-Whitney test used due to non-normal distribution of variable.

Descriptive analyses were done to explore if patients’ background differed by rural and Appalachian residence status (Tables 1 and 2, respectively). To summarize the main differences, patients from rural areas were more likely to be White, non-Hispanic (94.65% vs 85.17%) and insured through Medicare and Medicaid (47.52% and 21.68% vs 42.51% and 16.10%, respectively) than urban patients. Similarly, Appalachian patients were more likely to be White, non-Hispanic (94.60% vs 81.56%) and insured through Medicare and Medicaid (47.38% and 20.37% vs 40.73% and 15.42%, respectively) than non-Appalachian patients. Finally, rural patients were more likely to be seen in the gynecology and multidisciplinary clinics than urban patients (21.12% and 50.28% vs 14.69% and 46.83%, respectively), a pattern also seen for Appalachian compared to non-Appalachian patients.

### Responses to tobacco use screening and tobacco treatment offers

A history of tobacco use (i.e. current or former) was reported by 44.69% of patients, of which 21.75% reported current use. Among those with a history of tobacco use, 91.77% reported use of cigarettes, with smokeless tobacco being the second most common product (4.50%). Amid patients who reported current use, 76.65% declined the offer of tobacco treatment that would have triggered a referral to the ‘in-house’ tobacco treatment program. Of those who declined, 65.19% did so because they were not ready to quit, 27.29% because they desired to quit without assistance, and 6.73% because they were currently in treatment.

### Preliminary analyses of current tobacco use and response to tobacco treatment offers by residence

Descriptive analyses examined differences in key tobacco-related outcomes by rural and Appalachian residence. Patients from rural areas reported current tobacco use at a rate that was approximately six points higher than urban patients (25.74% vs 19.59%). In contrast, tobacco treatment referral decline was similarly high among rural and urban patients (77.37% vs 76.14%). The reason for declining was also similar across rural and urban patients: already in treatment (6.89% vs 6.62%), desire to quit without assistance (26.19% vs 28.08%), not ready to quit (66.33% vs 64.38%), and missing (0.60% vs 0.92%). Patients from rural areas reported current tobacco use at a higher rate (24.90% vs 16.86%), but there were similar rates of tobacco treatment referral decline (76.92% vs 76.03%) in rural and urban patients. The reason for declining was also similar across these groups of rural and urban patients: already in treatment (6.94% vs 6.26%), desire to quit without assistance (26.43% vs 28.28%), not ready to quit (65.87% vs 63.64%), and missing (0.77% vs 0.83%).

Appalachian patients also reported current tobacco use at a higher rate than non-Appalachian patients (25.50% vs 17.49%). In contrast, Appalachian patients declined tobacco treatment at similar rates as non-Appalachian patients (77.34% vs 75.51%) and the reason for treatment decline was also similar across groups: already in treatment (6.97% vs 6.33%), desire to quit without assistance (26.80% vs 28.12%), not ready to quit (65.50% vs 64.48%), and missing (0.74% vs 0.86%).

### Regression analyses of associations between key tobacco-related outcomes for both rural and Appalachian residence status

For the primary analyses, univariate and multivariable logistic regressions were completed (Tables 3 and 4). Adjusting for covariates, patients from rural areas were more likely to report current tobacco use than those from urban areas (OR=1.22; 95% CI: 1.12–1.34). The same was true for Appalachian residence, such that patients from Appalachian counties were more likely to report current tobacco use than non-Appalachian patients (OR=1.41; 95% CI: 1.28–1.54). Model goodness of fit for both binary logistic regressions were acceptable, as indicated by non-significant Hosmer–Lemeshow tests (p=0.346 and 0.432).

**Table 3:** Association of tobacco use status and response to tobacco treatment offer among US cancer survivors seeking care at a cancer center, with rural residence status, a cross-sectional analysis, 2019 (N=16839)

Variables	Current tobacco use OR (95% CI)	Treatment referral decline OR (95% CI)	Reason for treatment referral decline	
			Logit 1^a^	Logit 2^a^
			OR (95% CI)	OR (95% CI)
**Residence status**				
Urban ®	1	1	1	1
Rural	1.22 (1.12–1.34)*	0.87 (0.71–1.06)	1.01 (0.84–1.21)	0.94 (0.67–1.32)
p^b^	<0.0001	0.1754	0.9151	
**Clinic/disease site**				
Hematology ®	1	1	1	1
Breast	0.90 (0.76–1.07)	1.15 (0.77–1.70)	0.84 (0.59–1.20)	0.83 (0.42–1.63)
Gynecology	1.18 (1.01–1.38)*	1.30 (0.91–1.86)	1.38 (0.99–1.92)	0.63 (0.35–1.11)
Other	1.72 (1.52–1.94)*	0.87 (0.66–1.16)	0.69 (0.53–0.88)*	0.83 (0.49–1.39)
p	<0.0001	0.0620	<0.0001	
**Sex**				
Female ®	1	1	1	1
Male	1.73 (1.55–1.92)*	1.55 (1.23–1.96)*	1.23 (1.00–1.52)	2.07 (1.34–3.19)*
p	<0.0001	0.0002	0.0030	
**Race and ethnicity**				
White, non-Hispanic ®	1	1	1	1
Minority	0.81 (0.69–0.95)*	0.71 (0.50–1.00)	1.02 (0.72–1.43)	1.21 (0.62–2.39)
p	0.0104	0.0522	0.8492	
**Marital status**				
Married ®	1	1	1	1
Divorced/separated/ widowed	1.66 (1.49–1.85)*	1.05 (0.83–1.33)	0.87 (0.71–1.08)	0.99 (0.66–1.48)
Single	1.41 (1.26–1.59)*	1.08 (0.84–1.39)	0.87 (0.69–1.09)	0.85 (0.56–1.28)
p	<0.0001	0.8268	0.6038	
**Insurance status**				
Self-pay/other ®	1	1	1	1
Managed care organization	0.67 (0.51–0.89)*	1.01 (0.52–1.96)	0.74 (0.41–1.34)	0.22 (0.03–1.60)
Medicare	1.27 (0.96–1.69)	1.00 (0.51–1.93)	0.90 (0.50–1.61)	0.31 (0.04–2.28)
Medicaid	1.94 (1.46–2.57)*	0.87 (0.45–1.67)	1.06 (0.59–1.89)	0.19 (0.03–1.39)
p	<0.0001	0.6690	0.3240	
**Age tertiles** (years)				
<55 ®	1	1	1	1
55–67	0.80 (0.72–0.88)*	0.86 (0.68–1.08)	1.14 (0.93–1.41)	0.72 (0.50–1.05)
>67	0.30 (0.26–0.35)*	0.82 (0.59–1.15)	1.34 (0.98–1.81)	1.06 (0.55–2.05)
p	<0.0001	0.3541	0.0876	
**Distress tertiles**				
<1 ®	1	1	1	1
1–5	1.09 (0.97–1.22)	0.88 (0.66–1.17)	1.15 (0.91–1.46)	0.49 (0.30–0.80)*
>5	1.81 (1.63–2.02)*	0.62 (0.48–0.79)*	0.90 (0.73–1.12)	0.60 (0.38–0.96)*
p	<0.0001	0.0001	0.0010	
**Model goodness of fit** (H-L test)	0.3463	0.4316		

* Statistically significant.

a A proportional odds model was initially fit and a test of proportional odds was made. This test revealed that the proportional odds assumption did not hold for this cumulative logits model. Therefore, two separate sets of logits were formed by fitting a cumulative logits model where the first logit corresponds to the ‘log odds’ of not yet ready to quit versus want to quit on my own or already in treatment, and the second logit corresponds to the ‘log odds’ of not yet ready to quit or want to quit on my own versus already in treatment. These log odds ‘accumulate’ probability of ‘least desired to most desired outcome’. The model reported is the non-proportional odds/cumulative logits model.

b p-values from Type 3 Analysis of Effects Wald chi-squared statistic. H-L: Hosmer-Lemeshow. ® Reference categories.

**Table 4:** Association of tobacco use status and response to tobacco treatment offer among US cancer survivors seeking care at a cancer center, with Appalachian residence status, a cross-sectional analysis, 2019 (N=16839)

Variables	Current tobacco use OR (95% CI)	Treatment referral decline OR (95% CI)	Reason for treatment referral decline	
			Logit 1^a^	Logit 2^a^
			OR (95% CI)	OR (95% CI)
**Appalachian residence status**				
Non-Appalachian ®	1	1	1	1
Appalachian	1.41 (1.28–1.54)*	0.99 (0.80–1.22)	0.96 (0.80–1.16)	0.95 (0.66–1.37)
p^b^	<0.0001	0.909	0.912	
**Clinic/disease site**				
Hematology ®	1	1	1	1
Breast	0.89 (0.75–1.06)	1.13 (0.76–1.67)	0.84 (0.59–1.20)	0.83 (0.42–1.62)
Gynecology	1.16 (0.97–1.35)	1.27 (0.89–1.82)	1.39 (0.99–1.93)	0.62 (0.35–1.11)*
Other	1.68 (1.49–1.90)*	0.87 (0.65–1.15)	0.69 (0.53–0.89)*	0.83 (0.49–1.39)
p	<0.0001	0.071	<0.0001	
**Sex**				
Female ®	1	1	1	1
Male	1.73 (1.56–1.93)*	1.55 (1.23–1.95)*	1.23 (1.00–1.52)	2.06 (1.34–3.18)*
p	<0.0001	0.0002	0.003	
**Race and ethnicity**				
White, non-Hispanic ®	1	1	1	1
Minority	0.88 (0.75–1.04)	0.74 (0.52–1.05)	0.99 (0.70–1.40)	1.21 (0.60–2.44)
p	0.137	0.091	0.838	
**Marital status**				
Married ®	1	1	1	1
Divorced/separated/widowed	1.67 (1.50–1.86)*	1.05 (0.83–1.33)	0.87 (0.70–1.08)	0.98 (0.66–1.47)
Single	1.44 (1.29–1.62)*	1.09 (0.85–1.41)	0.86 (0.69–1.08)	0.84 (0.56–1.27)
p	<0.0001	0.783	0.585	
**Insurance status**				
Self-pay/other ®	1	1	1	1
Managed care organization	0.68 (0.51–0.90)*	1.01 (0.52–1.96)	0.74 (0.41–1.34)	0.22 (0.03–1.58)
Medicare	1.25 (0.94–1.66)	0.99 (0.51–1.91)	0.90 (0.50–1.61)	0.31 (0.04–2.25)
Medicaid	1.90 (1.43–2.52)*	0.86 (0.45–1.66)	1.06 (0.59–1.90)	0.19 (0.03–1.38)
p	<0.0001	0.647	0.325	
**Age tertiles (years)**				
<55 ®	1	1	1	1
55–67	0.80 (0.72–0.89)*	0.86 (0.68–1.08)	1.14 (0.93–1.41)	0.72 (0.49–1.05)
>67	0.30 (0.26–0.35)*	0.82 (0.59–1.15)	1.33 (0.98–1.81)	1.06 (0.55–2.06)
p	<0.0001	0.359	0.046	
**Distress tertiles**				
<1 ®	1	1	1	1
1–5	1.09 (0.97–1.23)	0.88 (0.66–1.17)	1.15 (0.91–1.46)	0.49 (0.30–0.80)*
>5	1.82 (1.63–2.02)*	0.61 (0.48–0.79)*	0.90 (0.73–1.12)	0.60 (0.38–0.96)*
p	<0.0001	0.0001	0.001	
**Model goodness of fit** (H-L test)	0.346	0.432		

* Statistically significant.

a A proportional odds model was initially fit and a test of proportional odds was made. This test revealed that the proportional odds assumption did not hold. Therefore, a fully non-proportional odds model was fit using two separate sets of logits where the first logit corresponds to the ‘log odds’ of not yet ready to quit versus want to quit on my own or already in treatment and the second logit corresponds to the ‘log odds’ of not yet ready to quit or want to quit on my own versus already in treatment. These log odds ‘accumulate’ probability of ‘least desired to most desired outcome’. The model reported is the non-proportional odds/cumulative logits model.

b p-values from Type 3 Analysis of Effects Wald chi-squared statistic. H-L: Hosmer-Lemeshow. ® Reference categories.

However, in separate models, neither rural nor Appalachian residence status were significantly associated with the decision to decline tobacco treatment (rural: OR=0.87; 95% CI: 0.71–1.06; Appalachian: OR=0.99; 95% CI: 0.80–1.22). For the ordinal outcome of reason to decline, a proportional odds model was initially tested, but the assumption of proportional odds was not met. Consequently, a non-proportional odds cumulative logits model was used, specifying two sets of logits: the first comparing ‘not yet ready to quit’ with ‘want to quit on my own’ and ‘already in treatment,’ and the second comparing ‘not yet ready to quit’ and ‘want to quit on my own’ with ‘already in treatment.’ These logits reflect the progression from least to most desired outcome. In separately tested models, neither rural (OR=0.94–1.01; 95% CI: 0.67–1.32) nor Appalachian (OR=0.95–0.96; 95% CI: 0.66–1.37) residence status was significantly associated with the preferred response.

## DISCUSSION

This study of electronic health record data from >16000 adult cancer patients examines the association between cancer survivors’ geographical residence and their report of current tobacco use and response to tobacco treatment offers. This study was done in the context of cancer care delivery, where identifying all individuals who use tobacco and providing evidence-based tobacco treatment can be considered the 4th pillar of care alongside surgery, radiation and chemotherapy^24^. Here, rural and Appalachian cancer survivors were found to report significantly higher levels of tobacco use than their urban and non-Appalachian counterparts, which is especially concerning because the overall rate of tobacco use in this sample (22%) is relatively high^6^. This mirrors national trends in clinical and non-clinical populations, underscoring the heightened vulnerability of rural and Appalachian residents for tobacco use and tobacco-related disease burden^25^. The persistence of tobacco use and related disparities tied to geographical residence may be attributed to a variety of demographic, socioeconomic, and cultural and societal factors, such as lower income, lower level of education, limited healthcare access, pro-tobacco social norms, and economic reliance on tobacco agriculture coupled with insufficient investment in tobacco prevention and control by regulatory agencies^18,19^. Notably, this study was conducted in a tobacco nation state – a region with a history of high smoking rates and significant tobacco industry interference in public health policies – further emphasizing the importance of addressing these disparities. The geographical isolation that is inherent to most rural and Appalachian areas and reduced preventive healthcare access, make it essential for cancer care and other specialty and tertiary care providers to take advantage of every clinical encounter by screening for tobacco use and providing tobacco treatment to all who report current use^26^.

This study also aimed to determine how cancer survivors from rural and Appalachian areas respond to offers of tobacco treatment. While the overall acceptance rate was low (18%), no differences were indicated based on rural or Appalachian residence status. The lack of group difference in this study contrasts with some prior literature, which has found that rural and Appalachian populations often experience lower levels of care utilization and help-seeking^27^. Prior observed discrepancies may, in part, be due to intrapersonal factors like cancer fatalism and cancer information overload, which are prevalent among rural residents^28^. In contrast, the current results align with a prior study’s finding that implementing an ‘opt-out’ tobacco treatment program increased treatment engagement among rural cancer survivors, which supports the broader conclusion that proactive, population-level interventions can effectively reach rural populations^25^. Another recent study highlighted how proactive, population-level offers of tobacco treatment can improve treatment access across diverse populations, specifically across Black and Hispanic cancer survivors^29^. Proactive outreach and populationlevel or universal offers of tobacco treatment may mitigate some of rural (and Appalachian) residents’ foremost barriers to engaging in treatment, as challenges with asking for help with a sensitive or stigmatized problem, scheduling appointments and finding suitable providers are inherently addressed at the system-level and the importance of cessation is clearly communicated by healthcare providers^30^.

The low overall acceptance rate of tobacco treatment observed in this study parallels relatively low utilization of such services in similar clinical populations^31^. Nearly two-thirds of the patients who declined reportedly did so because they were not ready to quit. Readiness to quit varies over time, and while most US adults who use tobacco would like to quit eventually^12^, at any given moment, most are not ready to do so^32,33^. In this way, study results converge with prior literature. However, contrary to hypotheses, there were no group differences by residential status regarding reasons for declining tobacco treatment. While unexpected because of multilevel barriers to cessation in rural and Appalachian regions, this could be viewed as a novel and encouraging result. It again highlights the need for cancer center system-level interventions that ensure everyone receives an offer of tobacco treatment, rather than relying on provider-level interventions that are subject to biases and assumptions tied to sociodemographic factors like geographical area of residence. At the same time, this study result also underscores the need to bolster readiness to quit among individuals receiving cancer care, potentially by implementing clinical strategies that have proven effective in other populations and care settings, such as tailored health education, nicotine replacement therapy sampling, and practice quit attempts^34^. These findings highlight the importance of comprehensive, system-wide approaches that offer cessation support to all cancer survivors while also addressing strategies to increase readiness to quit.

### Strengths and limitations

The study’s strengths include focusing on vulnerable populations and offering insights into a newly implemented tobacco treatment program. Importantly, tobacco treatment was equitably offered across patients from different sociodemographic backgrounds, and results demonstrated the potential to reach a high-risk population. This study’s large sample size enhances the reliability of the findings, particularly in evaluating the impact of factors such as geographical area of residence on tobacco use and tobacco treatment engagement outcomes.

However, a few methodological limitations of this study merit consideration for future research. First, the study was conducted at a single NCIdesignated comprehensive cancer center in the United States, which may limit the generalizability of the findings to other cancer care settings, regions, or countries with different healthcare systems or population characteristics. Second, the cross-sectional, observational design precludes causal inferences about the relationships observed. Third, tobacco use and readiness to quit were assessed through selfreported measures, which are subject to bias. Fourth, despite adjusting for several covariates, residual confounding by unmeasured factors cannot be ruled out. Fifth, patients might have misrepresented their tobacco use or reasons for declining tobacco treatment due to social desirability. Finally, the study relies on quantitative measures and does not explore the nuances of patients’ experiences and motivations through qualitative data, which could provide more insight into the unique barriers faced by rural and/or Appalachian cancer survivors.

## CONCLUSIONS

Tobacco treatment is a vital component of cancer care, and this study ultimately serves as a reminder that cancer survivors from rural and Appalachian regions stand to benefit from evidence-based care in the same manner as those from urban areas. Cancer care systems should continue quality improvement endeavors that increase accessibility to tobacco treatment and mitigate longstanding tobacco-related disease burden^35,36^.

## Data Availability

The data supporting this research are available from the authors on reasonable request.

## References

[1] Siegel RL, Giaquinto AN, Jemal A. Cancer statistics, 2024. CA Cancer J Clin. 2024;74(1):12-49. doi:10.3322/caac.21820 38230766

[2] American Cancer Society. Cancer facts & figures 2023. American Cancer Society; 2023. Accessed June 14, 2025. https://www.cancer.org/content/dam/cancer-org/research/cancer-facts-and-statistics/annual-cancer-facts-and-figures/2023/2023-cancer-facts-and-figures.pdf

[3] Vrinzen CEJ, Delfgou L, Stadhouders N, et al. A systematic review and multilevel regression analysis reveals the comorbidity prevalence in cancer. Cancer Res. 2023;83(7):1147-1157. doi:10.1158/0008-5472.CAN-22-1336 36779863 PMC10071818

[4] Brandenbarg D, Maass SWMC, Geerse OP, et al. A systematic review on the prevalence of symptoms of depression, anxiety and distress in long-term cancer survivors: Implications for primary care. Eur J Cancer Care (Engl). 2019;28(3):e13086. doi:10.1111/ecc.13086 31087398 PMC9286037

[5] Nipp RD, Shui AM, Perez GK, et al. Patterns in health care access and affordability among cancer survivors during implementation of the affordable care act. JAMA Oncol. 2018;4(6):791-797. doi:10.1001/jamaoncol.2018.0097 29596618 PMC5885177

[6] Arem H, Mama SK, Duan X, Rowland JH, Bellizzi KM, Ehlers DK. Prevalence of healthy behaviors among cancer survivors in the UNITED STATES: how far have we come? Cancer Epidemiol Biomarkers Prev. 2020;29(6):1179-1187. doi:10.1158/1055-9965.EPI-19-1318 32409489 PMC7778877

[7] National Comprehensive Cancer Network. Survivorship care for healthy living. National Comprehensive Cancer Network; 2024. Accessed June 14, 2025. https://www.nccn.org/patients/guidelines/content/PDF/survivorship-hl-patient.pdf

[8] National Center for Chronic Disease Prevention and Health Promotion (US) Office on Smoking and Health. The health consequences of smoking—50 Years of progress: a report of the Surgeon General. U.S. Department of Health and Human Services; 2014. Accessed June 14, 2025. https://www.ncbi.nlm.nih.gov/books/NBK179276/pdf/Bookshelf_NBK179276.pdf 32255575

[9] Warren GW, Cartmell KB, Garrett-Mayer E, Salloum RG, Cummings KM. Attributable failure of first-line cancer treatment and incremental costs associated with smoking by patients with cancer. JAMA Netw Open. 2019;2(4):e191703. doi:10.1001/jamanetworkopen.2019.1703 30951159 PMC6450325

[10] Oswald LB, Brownstein NC, Whiting J, et al. Smoking is related to worse cancer-related symptom burden. Oncologist. 2022;27(2):e176-e184. doi:10.1093/oncolo/oyab029 35641215 PMC8895733

[11] Cinciripini PM, Kypriotakis G, Blalock JA, et al. Survival outcomes of an early intervention smoking cessation treatment after a cancer diagnosis. JAMA Oncol. 2024;10(12):1689-1696. doi:10.1001/jamaoncol.2024.4890 39480450 PMC11528342

[12] U.S. Department of Health and Human Services. Smoking cessation. A Report of the Surgeon General. U.S. Department of Health and Human Services, Centers for Disease Control and Prevention, National Center for Chronic Disease Prevention and Health Promotion, Office on Smoking and Health; 2020. Accessed June 14, 2025. https://www.ncbi.nlm.nih.gov/books/NBK555591/pdf/Bookshelf_NBK555591.pdf

[13] Burris JL, Borger TN, Shelton BJ, et al. Tobacco use and tobacco treatment referral response of patients with cancer: implementation outcomes at a National Cancer Institute-Designated Cancer Center. JCO Oncol Pract. 2022;18(2):e261-e270. doi:10.1200/OP.20.01095 34185570 PMC9213199

[14] Levit LA, Byatt L, Lyss AP, et al. Closing the rural cancer care gap: three institutional approaches. JCO Oncol Pract. 2020;16(7):422-430. doi:10.1200/OP.20.00174 32574128

[15] Weaver KE, Geiger AM, Lu L, Case LD. Rural-urban disparities in health status among US cancer survivors. Cancer. 2013;119(5):1050-1057. doi:10.1002/cncr.27840 23096263 PMC3679645

[16] Bhatia S, Landier W, Paskett ED, et al. Rural-urban disparities in cancer outcomes: opportunities for future research. J Natl Cancer Inst. 2022;114(7):940-952. doi:10.1093/jnci/djac030 35148389 PMC9275775

[17] Cornelius ME, Loretan CG, Jamal A, et al. Tobacco product use among adults - United States, 2021. MMWR Morb Mortal Wkly Rep. 2023;72(18):475-483. doi:10.15585/mmwr.mm7218a1 37141154 PMC10168602

[18] Cardarelli K, Westneat S, Dunfee M, May B, Schoenberg N, Browning S. Persistent disparities in smoking among rural Appalachians: evidence from the Mountain Air Project. BMC Public Health. 2021;21(1):270. doi:10.1186/s12889-021-10334-6 33530976 PMC7856720

[19] Marshall JL, Thomas L, Lane NM, et al. Health disparities in Appalachia: the first report in a series exploring health issues in Appalachia. Appalachian Regional Commission; 2017. Accessed June 14, 2025. https://www.arc.gov/wp-content/uploads/2020/06/Health_Disparities_in_Appalachia_August_2017.pdf

[20] Roth AJ, Kornblith AB, Batel-Copel L, Peabody E, Scher HI, Holland JC. Rapid screening for psychologic distress in men with prostate carcinoma: a pilot study. Cancer. 1998;82(10):1904-1908. doi:10.1002/(sici)1097-0142(19980515)82:10<1904::aid-cncr13>3.0.co;2-x 9587123

[21] Rural-Urban Continuum Codes. Economic Research Service. Updated January 7, 2025. Accessed June 14, 2025. https://www.ers.usda.gov/data-products/rural-urban-continuum-codes

[22] Zuniga SA, Lango MN. Effect of rural and urban geography on larynx cancer incidence and survival. Laryngoscope. 2018;128(8):1874-1880. doi:10.1002/lary.27042 29238975

[23] Appalachian Counties Served by ARC. Appalachian Regional Commission. Accessed June 14, 2025. https://www.arc.gov/appalachian-counties-served-by-arc/

[24] Fiore MC, D’Angelo H, Baker T. Effective cessation treatment for patients with cancer who smoke-the fourth pillar of cancer care. JAMA Netw Open. 2019;2(9):e1912264. doi:10.1001/jamanetworkopen.2019.12264 31560380 PMC8295883

[25] Ramsey AT, Baker TB, Pham G, et al. Low burden strategies are needed to reduce smoking in rural healthcare settings: a lesson from cancer clinics. Int J Environ Res Public Health. 2020;17(5):1728. doi:10.3390/ijerph17051728 32155775 PMC7084618

[26] Lowy DR, Fiore MC, Willis G, Mangold KN, Bloch MH, Baker TB. Treating smoking in cancer patients: an essential component of cancer care-the new National Cancer Institute tobacco control monograph. JCO Oncol Pract. 2022;18(12):e1971-e1976. doi:10.1200/OP.22.00385 36343305 PMC10166433

[27] Nuako A, Liu J, Pham G, et al. Quantifying rural disparity in healthcare utilization in the United States: analysis of a large midwestern healthcare system. PLoS One. 2022;17(2):e0263718. doi:10.1371/journal.pone.0263718 35143583 PMC8830640

[28] Jensen JD, Shannon J, Iachan R, et al. Examining rural-urban differences in fatalism and information overload: data from 12 NCI-designated Cancer Centers. Cancer Epidemiol Biomarkers Prev. 2022;31(2):393-403. doi:10.1158/1055-9965.EPI-21-0355 35091459 PMC9035270

[29] Bates-Pappas GE, Schofield E, Chichester LR, et al. Universal tobacco screening and opt-out treatment referral strategy among patients diagnosed with cancer by race and ethnicity. JAMA Netw Open. 2024;7(4):e249525. doi:10.1001/jamanetworkopen.2024.9525 38648062 PMC11036136

[30] Hirko KA, Moore P, An LC, Hawley ST. Tobacco cessation motivations, preferences, and barriers among rural smokers: implications for optimizing referrals in clinical practice. AJPM Focus. 2022;2(1):100057. doi:10.1016/j.focus.2022.100057 37789934 PMC10546510

[31] Himelfarb-Blyth S, Vanderwater C, Hartwick J. Implementing a 3As and ‘opt-out’ tobacco cessation framework in an outpatient oncology setting. Curr Oncol. 2021;28(2):1197-1203. doi:10.3390/curroncol28020115 33799451 PMC8025814

[32] Burris JL, Feather AR, Pilehvari A, et al. Appalachian primary care patients’ quit readiness and tobacco treatment receipt. Am J Prev Med. 2025;68(2):396-401. doi:10.1016/j.amepre.2024.09.017 39343324 PMC11757061

[33] Wewers ME, Stillman FA, Hartman AM, Shopland DR. Distribution of daily smokers by stage of change: current population survey results. Prev Med. 2003;36(6):710-720. doi:10.1016/s0091-7435(03)00044-6 12744915

[34] Klemperer EM, Streck JM, Lindson N, et al. A systematic review and meta-analysis of interventions to induce attempts to quit tobacco among adults not ready to quit. Exp Clin Psychopharmacol. 2023;31(2):541-559. doi:10.1037/pha0000583 35771496 PMC10106992

[35] Cancer Center Cessation Initiative Diversity, Equity, and Inclusion Working Group Members. Integrating diversity, equity, and inclusion approaches into treatment of commercial tobacco use for optimal cancer care delivery. J Natl Compr Canc Netw. 2021;19(suppl 1):S4-S7. doi:10.6004/jnccn.2021.7091 34872050 PMC9040142

[36] Ostroff JS, Reilly EM, Burris JL, et al. Current practices, perceived barriers, and promising implementation strategies for improving quality of smoking cessation support in Accredited Cancer Programs of the American College of Surgeons. JCO Oncol Pract. 2024;20(2):212-219. doi:10.1200/OP.23.00393 37967292 PMC10911542

